# Barriers to the participation of men in reproductive health care: a systematic review and meta-synthesis

**DOI:** 10.1186/s12889-023-15692-x

**Published:** 2023-05-04

**Authors:** Robab Latifnejad Roudsari, Farangis sharifi, Fatemeh Goudarzi

**Affiliations:** 1 Nursing and Midwifery Care Research Center, Mashhad University of Medical Sciences, Mashhad, Iran; 2 Department of Midwifery, School of Nursing and Midwifery, Mashhad University of Medical Sciences, Mashhad, Iran; 3 Community-Oriented Nursing Midwifery Research Center, Shahrekord University of Medical Sciences, Shahrekord, Iran; 4 Social Determinants of Health Research Center, Yasuj University of Medical Sciences, Yasuj, Iran; 5 Department of Midwifery, School of Medicine, Yasuj University of Medical Sciences, Yasuj, Iran

**Keywords:** Reproductive health, Male involvement, Meta-synthesis

## Abstract

**Background:**

Despite emphasizing the importance and benefits of men's active engagement in reproductive health programs, their engagement in reproductive health care is low. Researchers have identified different barriers to men's avoidance of participation in various aspects of reproductive health in different parts of the world. This study provided an in-depth review of the hindrances to men’s non-participation in reproductive health.

**Methods:**

This meta-synthesis was conducted using keyword searches in databases including PubMed, Scopus, Web of Science, Cochrane, and ProQuest until January 2023. Qualitative English-language studies that investigated barriers to men's participation in reproductive health were included in the study. The critical appraisal skills program (CASP) checklist was used to assess the articles' quality. Data synthesis and thematic analysis were done using the standard method.

**Result:**

This synthesis led to the emergence of four main themes such as failure to access all inclusive and integrated quality services, economic issues, couples' personal preferences and attitudes, and sociocultural considerations to seek reproductive healthcare services.

**Conclusion:**

Healthcare system programs and policies, economic and sociocultural issues, and men’s attitudes, knowledge, and preferences, influence men's participation in reproductive healthcare. Reproductive health initiatives should focus on eliminating challenges to men's supportive activities to increase practical men's involvement in reproductive healthcare.

## Background

Reproductive health is a well-known family and social health component [1]. According to the definition of the World Health Organization, reproductive health means complete physical, mental, and social well-being in the functions and processes related to the reproductive system, not just the absence of disease and dysfunction or disability. Also, every person can have a good and safe sex life and freely decide about the time and manner of reproduction according to their desire [2]. From the mid-1990s until now, the importance and the benefits of men's active participation in reproductive health programs on the health of men, women, and children have been recognized and emphasized [3]. Despite the emphasis and importance of men's health in the definition of reproductive and sexual health, relatively few results for men's health have been obtained from this extensive reproductive health agenda [4]. In many studies, the role of men in reproductive health has been discussed based on women's health. Men effectively influence women's access to reproductive health care [5]. Commonly, Men make decisions about women's access to reproductive health care, money allocation for preventing the sexually transmitted diseases, family planning, and women's presence in antenatal and postpartum care, pregnancy and delivery care, transportation, nutrition, and child care [6].

The presence of women in reproductive health care, including family planning [7], antenatal care [8], safe motherhood [9], postpartum care [10], prevention of transition of HIV From mother to child(PMTC) [11], and sexually transmitted infections (STIs) [5] is often determined by their husbands. However, most men are not engaged in reproductive health care [7]. Franklin Ani (2015), reportedthe presence of men in reproductive health clinics was low (39.6%). He found that less than one-third of men (30.9%) participated in reproductive health-care [5]. Olayinka F.F et al. (2020) reported albeit most men were well aware of parenatal care, about 20% of them attended antenatal care with their partner, and (19.6%) participated in post-natal care [7]. Austin Wesevich et al. (2017) reported that male involvement in PMTC in Myanmar was 13% [12]. Also, Atuahene (2017) reported that most men (92.2%) did not accompany their wives to receive family planning services [9]. These quantitative studies provided numerical data about men's participation in reproductive health. These studies did not explain why men did not participate in reproductive health care [13]. Health system intervention and social, cultural, and economic factors are essential factors in the access and participation of people in reproductive and sexual health services [14]. We are witnessing different cultural, social, and economic contexts around the world that can make a difference in the access and participation of people, especially men. Thus, there is a need for a deep and detailed investigation of these factors and their impact on men's engagement in reproductive health services in different societies. Numerous qualitative research has explored men's participation in different aspects of reproductive health in various contexts [15-20]. They cited multiple reasons, such as reproductive health care as a feminine issue [15, 20], cultural issues [15, 18, 20], occupational matters [15, 18, 20], and economic issues [18, 20], were raised as male participation barriers to reproductive health.

Qualitative research helps to explore sentimental phenomena. Qualitative approaches provide the type of data that can help to understand participants' behaviors, feelings, and perceptions about the studied phenomena [21]. However, the small sample size has reduced the power of these studies to influence policymaking and planning. Another limitation of qualitative studies is the subjective interpretation of the data and the particular population studied, which challenges the transferability of the findings [13]. Synthesizing the data obtained from several qualitative studies is a way suggested by researchers to overcome the perceived limitations of qualitative approaches [22]. Meta-synthesis is a powerful method that examine qualitative studies and interprets and explains the phenomenon under study [22]. A systematic review of qualitative studies focuses on each unique phenomenon and its feedback. It identifies accurate evidence and summarizes it while appraising quality [23]. According toour knowledge a few studies have systematically reviewed men's participation in reproductive health through a meta-synthesis approach, including Louisa et al. (2014), who investigated men's views on contraception [24]. In this regard, the purpose of this study was to provide a comprehensive synthesis of views of women, men, and healthcare providers about barriers to men's engagement in reproductive health care that can help policy and planning to remove obstacles to male participation in reproductive health care. Thus, this study is looking for the answer to Why are men not involved in various aspects of reproductive health care?

## Methods

### Design

This qualitative meta-synthesis was conducted according to the methods described by Noblit and Hare (1988) [25], and the thematic analysis approach described by Braun and Clarke (2006) [26]. The Noblit and Hare methods consist of seven steps: determining the research question, selecting the research studies related to the research topic, evaluating the studies, deciding on how the studies relate, translating the studies to each other, synthesizing the translated concepts, and presenting the synthesized findings (Table 1). The thematic analysis approach described by Braun and Clarke has six steps, including data familiarity, generating primary code, searching themes by reviewing primary code, reviewing emerging themes, defining emerging themes, and preparing Report. According to Noblit and Hare, the first step to conducting a meta-synthesis is determining the aim and topic of the study, so the research question was developed: Why are men not involved in various aspects of reproductive health care?

**Table 1:** Steps of meta-synthesis according to Noblit and Hare (1988)

step	Description of steps
1	The beginning of the study: To determine the research question (the aim of the study)
2	Deciding on the choice of research studies related to the research topic: Considering the inclusion and exclusion criteria
3	Evaluation of studies: Reviewing the studies entered and examining the key concepts for data extraction
4	Deciding on how to relate studies: Creating code sheet and tables that include related concepts in the studies
5	Translating studies to each other: Comparing the key concepts of studies and translating them to each other
6	Synthesis of translated concepts: Creating a holistic concept
7	Presentation of synthesized findings

### Search strategy

The databases, including PubMed, Scopus, Web of Science, Cochrane, and ProQuest, were searched, systematically. The search was performed using the MESH terms including "Male Participation" OR "Men Participation "OR "Male Involvement" OR" Men Involvement" OR "Male Engagement" OR “Men Engagement" AND "Reproductive Health Care" OR "Maternal Health " OR "Sexual Health" OR "Family Planning" OR "Child Health" AND "Qualitative Study". The reference for included studies was searched manually. All the original qualitative studies from January 1994 until January 2023, focusing on the barriers to men's engagement in reproductive health care, which were published in the English language and their full text was available, were included in the study. All Studies with a quantitative design, areview, and meta-analysis articles were excluded.

### Study selection

**Fig. 1:**
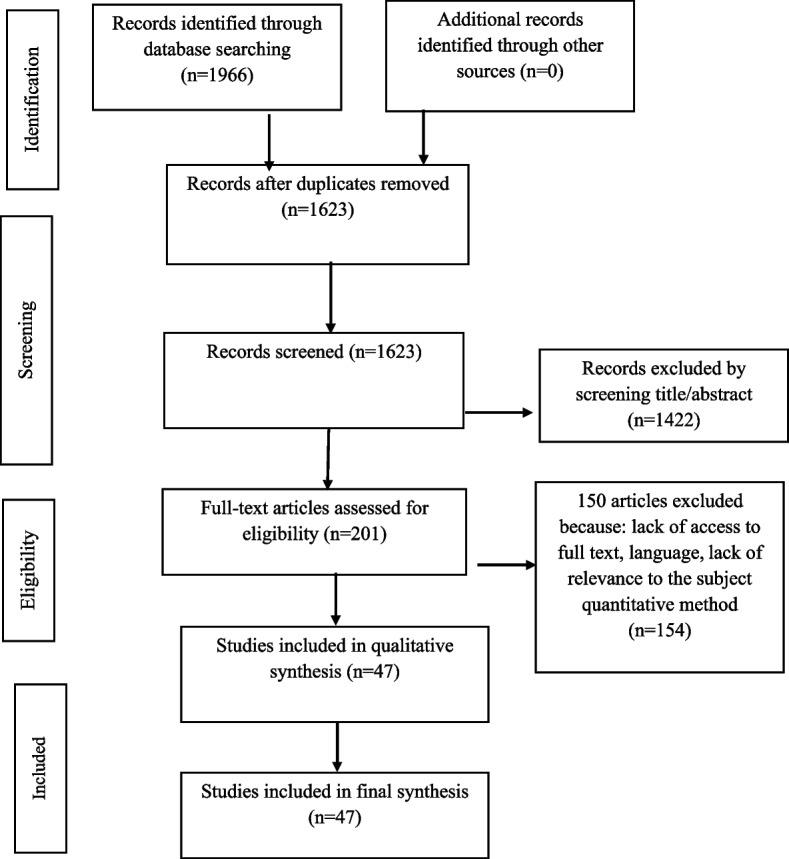
Preferred reporting items for systematic reviews and meta-analyses (PRISMA) flow diagram shows the s study selection process

The relevance of the articles with the research question was evaluated in several stages, such as the assessment ofthe title, the abstract, and the full text of the articles. This assessment was performed by two authors simultaneously. A total of 1966 articles were obtained from database searches. No article was found in the manual search. At each step of the screening, several studies were removed. The reasons for excluding the studies were the lack of relevance to the subject or the use of a quantitative approach. Finally, full-text screening was performed on 201 articles. Then, 47 qualitative articles related to the subject under study were selected. Figure 1 shows the process of study selection.

### Quality assessment

We considered the critical appraisal skills program tool (CASP version 2018) for appraising the selected studies [27]. Although the best way to evaluate the quality of qualitative articles is not agreed upon, in some meta-synthesis studies, the CASP checklist has been used to assess the article's quality [28, 29]. The quality of the articles was assessed by two authors separately. In case of disagreements between these authors, the opinion of the third author was discussed to reach a consensus. Table 2 shows the result of the included articles' quality assessment. No study was excluded from the meta-synthesis based on the score obtained from the quality assessment. Overall, the included articles had reasonable quality.

**Table 2:** The results of critical appraisal of included studies

Study	Research question	appropriateness of the methodology	appropriateness of the methodology	Recruitment strategy	Data collection	Reflexivity	Ethical issues	Data analysis	Findings	Contribution to knowledge	score
1 Nesane, K., S.M [15]	+	+	+	-	±	-	+	+	+	+	10
2 Teklesilasie, W. [16]	+	+	+	+	+	+	+	+	+	+	10
3 Gibore, N.S. [17]	+	+	+	+	+	+	+	+	+	+	10
4 Ongolly, F.K. [20]	+	+	+	+	+	+	+	+	+	-	9
5 Kwambai, T.K., et al. [30]	+	+	+	+	+	+	+	±	+	+	10
6 Nyondo, A.L., A.F., et a l [31]	+	+	+	+	+	+	+	+	+	+	10
7 Adongo, P.B., et al. [32]	+	+	+	+	±	-	+	±	+	+	8
8 Kabagenyi, A., et al. [33]	+	+	+	+	+	±	+	±	+	+	9
9 Dral, A.A., et al. [34]	+	+	+	+	±	-	+	+	+	+	9
10 Adejoh, S.O., A, et al. [35]	+	+	+	+	+	-	+	±	+	±	8
11 Auvinen, J., et al. [35]	+	+	+	+	±	±	+	±	+	+	8
12 Mohlala, B.K., S. et al. [36]	+	+	+	+	+	-	+	±	±	-	7
13 Falnes, E.F., et al. [37]	+	+	+	±	±	+	+	±	+	+	9
14 Gill, M.M., et al. [38]	+	+	+	+	±	-	+	+	+	±	8
15 Jungari, S.et al. [39]	+	+	+	±	±	-	+	±	+	±	7
16 MirzaiiNajmabadi, K., et al. [40]	+	+	+	+	+	±	+	+	+	+	10
17 Dovel, K., et al. [41]	+	+	+	+	±	-	+	±	+	+	8
18 Kashaija, D.K [42]	+	+	+	+	+	+	+	+	+	+	10
19 Ladur, A.N.et al. [43]	+	+	+	+	+	+	+	+	+	+	10
20 Kaida, A., et al. [44]	+	+	+	±	±	±	+	±	+	±	8
Maluka, S.O., et al. [45]	+	+	+	+	±	+	+	±	+	+	9
McEvoy, R., et al. [23]	+	+	+	+	±	+	+	+	+	+	10
Lowe, M., [46]	+	+	+	+	±	+	+	+	+	+	10
Gibore, N.S., et al. [8]	+	+	+	+	+	+	+	+	+	+	10
Aborigo, R.A., et al. [19]	+	+	+	+	+	+	+	+	+	+	10
Mkandawire, E., et al. [47]	+	+	+	+	±	+	+	+	+	±	9
Firouzan, V., et al. [18]	+	+	+	+	+	+	+	+	+	+	10
Greenspan, J.A., et al. [48]	+	+	+	+	±	+	+	+	+	+	10
Ganle, J.K. [49]	+	+	+	+	+	+	+	+	+	+	10
Sakala, D., et al. [50]	+	+	+	+	+	-	+	+	+	+	9
Gopal, P., et al. [51]	+	+	+	+	±	+	+	+	+	+	10
Shahjahan, M, et al. [52]	+	+	+	±	±	-	±	±	+	±	6
Sharma S et al. [53]	+	+	+	+	±	-	+	+	+	+	9
Vermeulen, E., et al. [54]	+	+	+	+	±	+	+	+	+	+	10
Sharma, S. et al. [55]	+	+	+	+	+	+	+	+	+	+	10
Dumbaugh M et al. [56]	+	+	+	+	+	-	+	+	+	+	8
Yeganeh N et al. [57]	+	+	+	+	+	-	+	+	+	+	8
Willcox ML et al. [58]	+	+	+	+	+	+	+	-	+	+	9
Mapunda B et al. [59]	+	+	+	+	+	-	+	+	+	+	9
Okafor IP et al. [60]	+	+	+	+	+	-	+	+	+	+	8
Sakala D et al. [50]	+	+	+	+	+	+	+	+	+	+	9
Forbes F et al. [61]	+	+	+	+	+	+	+	-	+	+	9
Davis J et al. [3]	+	+	+	+	+	-	+	+	+	+	8
Sharma V et al. [53]	+	+	+	+	+	+	+	+	+	+	9
Koffi TBet al [62]	+	+	+	+	+	+	+	+	+	+	10
Dychtwald DK et al. [63]	+	+	+	+	+	+	+	-	+	+	8
Shongwe P et al. [64]	+	+	+	-	+	-	+	+	+	+	8

### Data extraction

The next step was to read the full text of each article identified for inclusion in the review and to extract the pertinent data using a standardized data extraction form. Data were extracted in collaboration with two authors (F.G and F.S). The extracted data included the author's name, year of publication, the purpose of the study, study population, country of study setting, study design, number of participants, method of analysis, the main focus of the studies, and study's main findings (Table 3).

**Table 3:** Summary of characteristics of the articles included in the meta-synthesis

Authors, Publication date and Setting	Purpose	Design	Field of study	Sample’s characteristics (Men or Women)	Type of data analysis	Method of data collection	Findings	Sample size
Nesane, K., S.M [15] (2016) Guatemala	Exploring men's views about their participation in the maternal health center	exploratory descriptive	Maternal healthcare	Husband of women who became pregnant 2 years ago	thematic analyze	individual interviews	Them: Maternity care issues are viewed as a Femininity domain. Three sub-themes of culture and involvement in childbirth, job status of male partners, and the unwillingness of men to involvement in maternal health issues	15
Teklesilasie, W. [16] (2020) Ethiopia	to determine the barriers of men participate in maternity health care	exploratory descriptive	maternity care	Men who have had a child in the last 5 years	thematic analyze	individual interviews,face-to-face discussions	parturition is a natural procedure Pregnancy and childbirth is a women's issue Prefer to receive TBA care spouse participation in maternity care as a new idea	102
3 Gibore, N.S [17] (2020) Tanzania	Determining social perspectives on barriers that potentially prevent men from participating in pregnancy care	qualitative approach	maternal health care	Couples with children 2 years and younger, society leaders, Rural health workers, Health professionals	thematic analyze	focus group discussions in-depth interviews	in pregnancy care Behavior based on gender expectations set in culture in pregnancy care, Extramarital pregnancy, Fear of HIV testing, Economic and family instability-unawareness-Health system issues	66
4 Ongolly, F.K [20] (2019) western Kenya	Determining barriers to the presence of men in antenatal and postpartum care	mixed methods	Antenatal care postnatal care	Men who had children in the past year key informant	thematic analyze	focus group discussions	Barriers to men's participation included cultural issues, economic issues, health system issues. Sub-themes included: Maternal health is a women's issue, existence the traditional maternal care (cultural issues), men's jobs, low income, care's costs (economic issues) and lack of services related to men, time-consuming services, health care providers' perspective, lack of space For men, lack of privacy in the centers (health system)	44
Kwambai, T.K., et al. [30] (2013) Kenya	exploring the Men's perceptions of antenatal Care	qualitative approach	antenatal and childbirth care	married men	thematic analyze	focus group discussions	Supporting pregnant women as a female duty, Men as the provider, Men as decision maker, The negative attitude of health providers about male participation, Unfriendly structure of maternal care centers for couples	68
6 Nyondo, A.L., A.F.,et al. [31] (2014) Malawi	Determining of promoting and hindering factors for men's participate in preventing of transition of HIV from mother to child	exploratory qualitative study	preventing of transition of HIV from mother to child	men AND women AND Medical Assistant	thematic analyze	Focus Group discussion face to face Interviews	Unawareness of men about PMTCT, socio-economic issues, communication issues Being shy about issues related to women, unwanted pregnancy or extramarital Pregnancies, Fear of finding out of his HIV status, Reluctance to communicate with the service, Issues related to health facilities, peer effects and cultural Issues	41
7 Adongo, P.B., et al. [32] (2014) Ghana	Exploring the community perceptions about vasectomy and its influences on vasectomy acceptance	qualitative approach	vasectomy	Health workers and health nurses and community members including men, women and health volunteers	thematic analyze	focus group,interviews	Considering Vasectomy as an action against the will of God and subject to the death penalty or accountability on the Day of Judgment, Perception of vasectomy as castration leads to weakening of men in female sexual satisfaction, Concerned about the negative side effects of vasectomy on men, Using the alternative medicine as contraception	148
8 Kabagenyi, A., et al. [33] (2014) Uganda	Exploring women's and men's perspectives on components that prevent men from supporting and using the contraception methods	A cross-sectional qualitative study	contraceptive uptake and reproductive health	Men, women, Government and society leaders	thematic analyses	Focus group interviews	Components that prevent men from participating include: negative Side effects of feminine methods of contraceptives that cause sexual dysfunctionRestriction of available manly contraceptive methods, considering family planning as a femininity issue through perceived gender norms and traditional interaction in reproductive health care, The tendency to have a large family is hindered by the distance between births, Men's concern about their spouse's extramarital sex after taking contraceptives	162
Dral, A.A., et al.[34] (2018) Malawi	Determining the effective components on participation of men in family planning	qualitative study	Family Planning	men, women and health surveillance assistants	Inductive content analysis was performed	semi-structured interviews	Components that motivate people's health behaviors, Gender norms governing relationships, health behavioral proficiencies, Awareness about health behaviors, socio-economic, socio-economic components	23
Adejoh, S.O., A, et al. [65] (2017) Nigeria	Determining the components that influenced men's involvement in maternity health issues	A cross-sectional qualitative study	Maternal Health	married men	thematic analyze	in-depth interview	Health care price, Downturn, and job responsibility as the Cause of non-active involvement of men in antenatal care	30
Auvinen, J., et al. [35] (2013) Zambia	Exploring men's views on the components that prevent participating them to the program of preventing the transit of HIV from mother to child	qualitative descriptive study	preventing the transit of HIV from mother to child	men	Described Content Analysis by Miles and Huberman (1994)	in-depth interview	The barrier of men involvement: Conditions such as poverty, refugee, lack of supportive preparations, and the prevailing culture of prenatal care, passivity, unawareness, marital disagreement, stigma caused by HIV-related conditions, and cultural issues, including patriarchy and religious beliefs prevented men from participating	21
Mohlala, B.K., S.et al. [36] (2012) South Africa	Exploring men and pregnant women's experiences, feelings, beliefs, attitudes, and about men's participation in antenatal care	qualitative studies	Antenatal care	pregnant women and men	Inductive(Goss & Leinbach, 1996) and Deductive (White & Thomson, 1995) analysis	Focus group discussions	unawareness about the male role in antenatal care, Employment Social reasons, Facility and staff attitudes, and Cultural reasons were Obstacles to the presence of men in the antenatal clinic	60
Falnes, E.F., et al. [37] (2011) Tanzania	Determining the acceptance factors of the preventing of transition of HIV from mother to child program and identifying the challenges associated with male participation	Mixed methods	preventing of transition of HIV from mother to child	Women and Men and health personnel	Thematic content approach, (Graneheim and Lundman)	focus group and interview	The main barriers reported: Women were not allowed to ask their husbands for HIV testing. The antenatal Clinic, where HIV testing is performed there, was known as the female environment that men had no presence there	46
Gill, M.M., et al. [38] (2017) Congo	Determining factors affecting the presence of men in antenatal care and identify interventions that potentially improve men's participation	qualitative study	Antenatal services	Men and Women and public health and medicine key informants	thematic analyze	focus group discussions and in-depth interview	Common obstacles include: Lack of time due to job issues, The unfriendly environment of the clinic for men and couples, Considering pregnancy as a female domain and Men were afraid of testing for HIV	38
Jungari, S.et al. [39] (2019) India	Evaluation of men's participation in antenatal care, childbirth, postpartum care (PNC), housework, and food supply	mixed-method	maternity care	Women with children under 2 years old men whose wives gave birth health care providers village head trained/untrained dais	thematic analyze	Interviews focus group	Men believed that their presence in maternal care was not necessary and also they believed that maternity care is a feminine issue that prevented them to participate in maternity care	385
MirzaiiNajmabadi, K., et al. [40] (2019) Iran	Determining the barriers to men's participation in reproductive and sexual health education	conventional content analysis	Sexual and Reproductive Health Education	Men, religious scholars, health professionals, officials of health organizations	conventional qualitative content analysis	individual in-depth semi-structured interview	perceived threat less than usual, reluctance to learn, socio-cultural taboos, inadequate knowledge of family and Poor performance in family, policy barriers, executive barriers, and health systems deficiency under three topics including individual problems, socio-cultural issues, and structural problems as barriers to participation Men were identified	34
Dovel, K., et al. [41] (2020) Malawi	Investigating the systematization of health institutions and determining the men s perspective of participating in HIV testing	mixed methods ethnography	HIV testing	healthcare workers national key informants rural health facilities observational journals	deductive and inductive technique Based on technique Atlas. Tivol version 6	In-depth interviews in direct observation	Gender expectations intertwined with health organizations at three levels: organizational policy, organizational performance, and structural	29
Kashaija, D.K [42] (2020) Tanzania	Exploring the men's perceptions and experience of their spouse support in maternity care	Qualitative descriptive study	supporting the wives during childbirth	Men	qualitative content analysis	in-depth interview	Problems in transferring spouses to health centers due to poor road infrastructure, lack of support for men's accommodation in health centers, the financial instability of men, the attitude of the health care provider prevented men from participating to support their wives	9
Ladur, A.N. et.al [43] (2015) South Africa	Exploring the perspective of men, women, and health care providers about men's participation in preventing of transition of HIV from mother to child program	exploratory qualitative	preventing of transition of HIV from mother to child	Men Women HIV-positive couples service providers	Two methods: summative Content and Thematic Analysis	focus group interviews	Fear of stigma, Staff shortages, negative attitudes of health providers, tiny space of health centers, worrying about privacy, Time consuming services, and discomfort from being in the feminine environment of the health centers prevented men from participating in PMTCT services	25
Kaida, A., et al.[44] (2005) Uganda	Exploring men's perspectives about family planning and determining how they tend to involve in the family planning program	Qualitative study	family planning	key informants married men	Thematic analysis	Interviews focus group discussion	Inadequate information, misconceptions, fear of side effects of contraceptive methods, insufficient access to family planning services, mistrust of service providers, Distrust between couples, failure to consult between the couple, and cultural-religious issues were barriers for participation of men in family planning programs.	31
Maluka, S.O [45] (2018) Tanzania	Determining context-based perspectives on men's participation in maternity care	Qualitative study	pregnancy and childbirth care	Women who were pregnant or gave birth in the past year, men who accompanied their spouse to care center, health center staff, society leaders, social assistant, traditional birth service providers	Thematic analysis	In depth interviews	The determining gender roles in the family, fear of HIV testing, and inappropriate health centers environment for men's presence prevented them from participating	53
McEvoy R. et al. [23] (2018) Burkina Faso	Exploring the viewpoints of men and women on hindrances to use of family planning method	qualitative study	family planning	men and women opinion leaders adult men and Married women	constant comparative technique	focus groups in-depth interviews	The barriers to men's participation in family planning included the negative attitudes of men, ignorance of modern methods of contraception, misconceptions about modern methods of contraception, concerns about side effects of contraceptive methods, cultural norms, and social preferences for having large families	52 20 focus groups
Lowe, M [46]. (2017) Gambia	Determining some basic socio-cultural issues effective on men's participation in maternity health issues	qualitative study	maternal health	Men, Traditional birth attendant	thematic analysis	focus group discussion in-depth interviews	Maternity issues were perceived as female issues. Competition of job responsibilities with issues related to maternity care. Competition between the wives of polygamous men, fear of stigma	56
Reuben Mahiti, G, et al. [66] (2017) Tanzania	Determining men's viewpoints of cultural actions during postnatal care	qualitative study	Postnatal care	man	content analysis (Griesheim and Lundman)	focus group discussion	Embarrassing about participating in pregnancy care, Belief in Bing the femininity of reproductive issues, Bing unusual the Accompanying of men with their spouse in reproductive health clinics socially were barriers of male participation in reproductive health	93
Aborigo, R.A., et al. [19] (2018) Ghana	Determination of the causes of men's resistance to the acceptance to play the active role in maternity care and their effects in the decision-making process for emergencies	qualitative study	maternal health	health staff society leaders	content analysis	focus group interviews	Men believe that accompanying spouses in maternity care are Unnecessary practiced. Men's involvement in maternity cares an embarrassing practice. Men believe that expressing affection to the spouse is embarrassing. Inappropriate clinical infrastructure to the presence of men	136
Mkandawire, E. et al. [47] (2018) Malawi	Determining the perception of rural people about the concept of men's participation in maternal and child health. Exploring the effective factors for men's participation in maternal and child care	qualitative study	maternal and child health	Informants community members	thematic analysis	focus group interviews	Sociocultural issues, fear of social stigma, costs of male participating in antenatal care prevent men from participating in antenatal care	70
Firouzan, V., et al. [18] (2019) Iran	Explore the factors that prevent men from participating in prenatal care	qualitative research	Perinatal care	Pregnant women, women who have given birth recently spouse of pregnant women Health staff	conventional content analysis	interviews, focused group	Cultural issues, personal issues, personal preferences, interpersonal issues, infrastructure issues in the health system, and socio-economic issues were the main obstacles for men	45
Greenspan, J.A., et al. [48] (2019) Tanzania	Determining factors to increase men's participation in maternal and infant health services	qualitative research	Maternal and newborn health	Men	inductive analyzed	in-depth interviews	Factors associated with men's non-participation in maternal and infant care were inadequate knowledge, preference for economic activities, costs associated with care, and limiting the presence of men in health centers due to the policies governing these centers	27
Ganle, J.K., et al. [49] (2015) Ghana	To determine the limiting and encouraging factors of men's participation in maternal care	qualitative research	maternal healthcare	adult men and his wife and key informants	thematic analysis	Focus group interviews	Male-dominated gender roles in society and related issues, cultural issues and practices, the financial burden of accompanying women in maternal care, and the structure of health services such as negative attitudes of health personnel, small space, inadequate working hours were factors that limited men's participation in maternal care	80
Sakala, D., et al. [50] (2021) Malawi	Exploring the main factors preventing men from participating in antenatal care and determining the importance of facilitator of male participation	qualitative study	Antenatal care including HIV testing	men and women	Thematic analysis	focus group interviews	The men's preference for economic activities, fear of being seen by their friends, discomfort with being in the feminine environment of center, health center environment issues, and providing HIV related services along with other maternity services caused reducing the men's presence in antenatal services	60
Gopal, P., et al. [51] (2020) Uganda	Exploring the perspectives and experiences of stakeholders on male participation in reproductive health by considering economic and political factors	qualitative study	reproductive health	Men participating in maternity health care, their spouses, organizations, and people involved in male participation in reproductive health	thematic analysis	focus group and interviews	Lack of accurate conversion of policies into action, Problems with resources in the health system and lack of skills to involve men in her reproductive health care, ‘inadequate participation by key actors’, and ‘types of dissemination’	
Shahjahan, M, et al. [52] (2006) Bangladesh	Exploring the men's awareness and viewpoint on reproductive health and how to increase men's participation	qualitative study	Reproductive health	Men	Dot tell	focus group	Men's lack of motivation to participate in reproductive health care, Feeling no need for reproductive health services Not encouraging men to participate in reproductive health services Lack of understanding of women's reproductive health issues due to interaction problems between couples Lack of companionship with the spouse in health services due to the prevailing culture in the community Reluctance to associate with health workers	38
Sharma V,et al. [53] (2019) Nigeria	To investigate engagement of men in maternal and infant health	qualitative study	maternal and newborn health	Husbands community leaders community health workers	Thematic analysis	Interviews focus group	Inadequate knowledge, Men's gender viewpoint on issues associated with maternity care, fear of mockery, and a negative viewpoint of health's staffs, led to the limited participation of men in maternity care	82
Vermeulen, E., et al. [54] (2016) Tanzania	Exploring the rural men's Perception, perspective, and behavior on participating in maternity care	Mixed method approach	pregnancy	men and health worker	Don’t tell	focus group interviews observation	A traditional gender perspective on pregnancy and maternity care, inadequate knowledge, insufficient access to prenatal care due to unavailable facilities to join the pregnancy care, and the negative experience of attending health centers	112
Sharma, S.et al. [55] (2018) Nepal	Exploring the gender attitudes of health workers and teachers about the components involved in men's participation in reproductive health care	qualitative study	reproductive health	Male teachers Health professionals of the health post	content analysis	focus group and interviews	The institutionalized socio-cultural norms, lack of training about reproductive health, misconception, predomination of women as health care workers in health centers hindered the participation of men in reproductive health	20
Dumbaugh M [56] (2014) Ghana	Exploring perceptions, attitudes, and barriers of male participation in newborn care in Ghana	qualitative study	Newborn care	Men and women who recently became parents	Based on the guidelines for reviewing qualitative research	focus group and interviews	Barriers identified to increase male participation in newborn care include factors related to the division of labor and space based on gender and generation	59
Yeganeh N [57] (2017) Brazil	Exploring the barriers and facilitators of men's participation in prenatal care and HIV counseling in Brazil	qualitative study	prenatal care and STI	Men who did and who did not Meet whose wives and infants in the postpartum ward	Thematic analysis	interviews	Stigma against HIV-positive people and the conflict between men's work and pregnancy care planning were the main barriers to men's participation	35
Willcox ML [58] (2021) Uganda	exploring the barriers and facilitators of couples counseling programs on postpartum family planning and prenatal counseling in Uganda	qualitative study	family planning	Women who attend antenatal or postnatal clinics Men who attended the health facility Health workers who work in reproductive health care clinics	thematic analysis	Interviews and focus groups	The contradiction between work and attendance at the family planning clinic, transportation costs, and the feeling of stigma caused by attendance at the reproductive health clinics are some of the obstacles to the participation of men in the family planning counseling program after childbirth	338
Mapunda B [59] (2022) Tanzania	Exploring the men's views on participation in prenatal care and determining the factors involved in their participation	Mixed method approach	Antenatal care	Men whose female partners attended the antenatal clinic	Thematic analysis	focus group	The cultural factors and gender perspective, inadequate knowledge about antenatal care, factors beyond men's control, conflict in relationships between couples, and obstacles related to the structure of antenatal service were five factors that were identified as barriers to men's participation	18
Okafor IP [60] (2022) Nigeria	Assessing the participation of men in maternal and child health and Exploring the barriers and predictors of men's participation in maternal and child health	mixedmethod approach	maternal and child health	Adult men who have at least one child under the age of 5 living together in the family	Thematic analysis	focus group discussion	Socio-cultural factors, lack of time for participation, fear of being stigmatized, the views of health workers, and costs related to maternal and child health care were among the most important barriers to men's participation	11
Sakala D [50](2021) Malawi	Exploring the barriers and facilitators of men's participation in antenatal care and HIV testing	qualitative study	antenatal care and STI	women who attend ANC for the first time and their partners	Thematic analysis	ocus group discussion and interview	Social norms, gender perspective of antenatal care, peer's perspective, fear of HIV testing, and conflict between job commitment and participation in antenatal care were the most important obstacles to men's participation in antenatal care	62
Forbes F [61] 2021 Australian	Exploring the experiences, attitudes, and beliefs of recent Ethiopian immigrant families living in Australia about men's participation in perinatal care	qualitative study	perinatal care	Ethiopian migrant men and women living in Australia and attended maternity care	Thematic analysis	interviews	Employment in the form of paid work, Restrictions the paternity leave, prioritizing earning over participation in care, and cultural and social issues governing families were important obstacles to men's participation in prenatal care	13
Davis J [3] (2016) Pacific	Explaining the perspective of maternal and child health professionals about the benefits, barriers, and approaches to increasing men's participation in maternal and child health services	qualitative study	maternal and child health	Senior professionals in health sectors, UN agencies, hospitals, universities, regional NGOs	Thematic analysis	interviews	Social norms and cultural issues, difficulties in communicating the health system with couples before pregnancies, the physical environment of health service centers, the perspective of health center staff, and their heavy workload, were the important challenges of men's participation in maternal and child health care	17
Sharma V [53] (2019) Nigeria	Exploring men's participation in mother and child health	qualitative study	mother and child health	Men whose wives or children had complications or died Community leaders Health providers People who were present when complications occurred for the mother and baby	Interviews and focus group discussions	Thematic analysis	The absence of female health workers, lack of medicine and equipment, distant health service centers, attitudes and behaviors of health providers, and, misbehavior of health workers were identified as barriers to men's participation	58
Koffi TB [46] (2018) Togo	Exploring the perspectives to improve men's participation in future family planning programs in Togo	A qualitative study	Family planing	Married men include: professional workers, killed and unskillednskilled	focus group discussions	Thematic analysis	Fear of the side effects of contraceptives, concern about the potential threat of fertility, concern for the potential health risks of mother and child following the use of contraceptive methods, and misbeliefs about the use of contraceptive methods were the reasons why men avoid participating in family planning	72
Dychtwald DK [63] (2021) USA	Exploring men's experiences and perspectives about supplementary nutrition programs for women, infants, and children	qualitative study	mother and child health	Couples who enrolled in the supplementary nutrition program for mothers, infants, and children	thematic analysis	interview	Barriers to men's participation in WIC were potential threats to men's pride, fear, and distrust of welfare programs, fear of mandating the participation of men, the unknown role of men in the WIC program, men feeling of being ignored, conflict of working hours of the WIC program with men's working hours, disclosure concerns arising from the relationship between the administrative environment and the WIC program, and Ignoring men in program naming	16
Shongwe P [64] (2019) Eswatini	exploring the understanding of men's perspective on the acceptance of vasectomy and understanding the causes of their poor participation in vasectomy	Qualitative study	Family planing	The sample includes single and married men living in urban and rural areas	thematic analysis	focus group discussion	Ignorance, cultural beliefs, social issues, false beliefs were the barriers to accepting vasectomy	54

### Data synthesis

For the synthesis in this study, a combination of the classical meta-synthesis or meta-ethnography was adopted by Noblit and Hare (1988) [25], as well as Brown and Clark's (2006) thematic analysis approach [26], was used. This combination has been successfully used in previous reviews [28, 29]. The approach described by Noblit and Hare [1988] focuses on the reciprocal translation, reliable synthesis, and lines of reasoning. Reciprocal translation analysis identifies concepts in each study, compares these concepts with those of other studies, and selects a comprehensive meaning that includes other similar meaning [26]. Although the Noblit and Hare approach are explained the seven steps of meta-synthesis and translations, the practical process of meta-synthesis of this approach is not clarified clearly Ed [67]. Studies have criticized this lack of expressiveness [67, 68]. It has been discussed that sometimes it is impossible to implement a cross-translational study. For example, an approach such as "first identified translated first "or "oldest paper translated first" can be challenging in meta-synthesis especially, when there is a lot of data and different perspectives. In addition, it is difficult to ensure that the quality of an article that initiates the translation process is better than others. Sometimes it is difficult to agree on a high-quality study [69, 70]. Sometimes, the translation process starts with an article, but that article may be conceptually weak, and this issue can affect subsequent translations [29]. To better manage the data and clarify the analysis process, we used the thematic analysis approach to modify steps 3–6 of the meta-synthesis adopted by Noblit and Hare approach (1988).

**Fig. 2:**
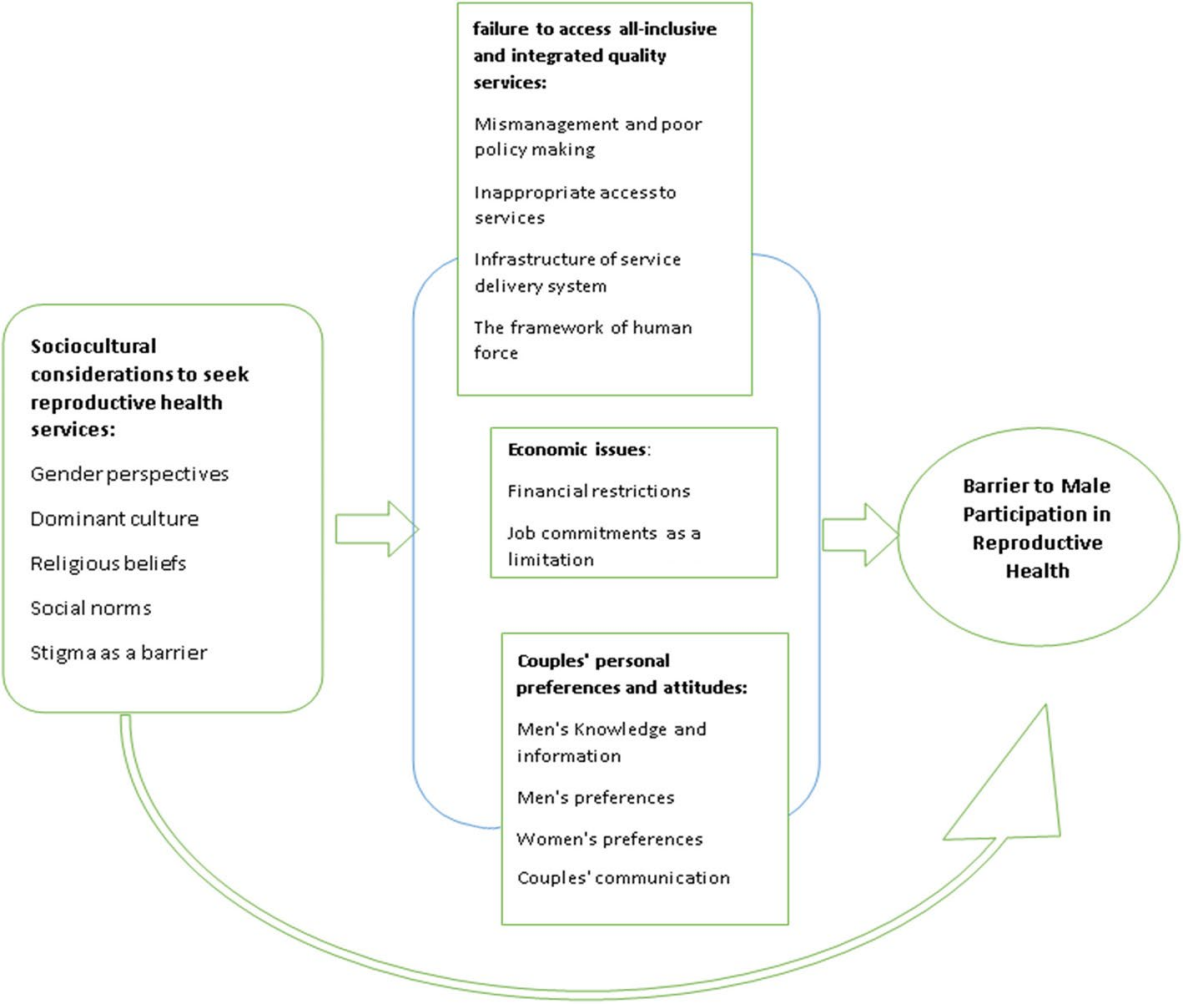
The illustrated conceptual model of the relationship between the identified barriers to men's participation in reproductive health care

Thematic analysis was conducted based on Brown and Clark's approach (2006). It is a six-step process that focuses on examining themes in the text. So after entering the results of the studies in the software MAXQD (version 10), the researchers read the text several times to understand the meanings and patterns of the data. After getting acquainted with the data, the process of coding started. The initial codes were written by describing the label and determining its location (referenced). A list of described codes was prepared. The meaningful sections were identified by a systematic method. Then the data was reduced to mini-meaning units. The extracted codes were frequently compared with each other. The extracted initial codes were reviewed by the third author (R.L.R). The similar extracted codes (concepts) were categorized into subthemes. Then the main theme that covered sub-themes emerged. The main theme was evaluated and condensed in terms of meaningfulness, relevance with sub-themes, and relevance with the concept of the included studies. This step was conducted with the participation of all authors, and a consensus was reached through discussion. Finally, the themes were defined and interpreted. Then the synthesis of the data under each theme was completed that was supported by the evidence from included articles. Finally, the”line of argument” was conducted to clarify the linkages between the extracted concepts from the synthesis. Developing the theoretical insights helps understand the barriers that led to the lack of men's engagement in reproductive health care (Fig. 2).

## Results

Among 1966 articles, 47 studies met the inclusion criteria and were included in the synthesisThe included studies contain of data from 3051 participants. This data were collected using focus groups and in-depth semi-structured interviews. Study participants included men, women, health professionals, and society leaders. These studies examined various aspects of reproductive health care.

### Characteristics of included studies

These studies were conducted in 24 countries, such as Tanzania (9 studies), Malawi (6 studies), Ghana (4 studies), Uganda (4 studies), Nigeria (4 studies), South Africa (2 studies), Iran (2 studies), western Kenya (1 study), Guatemala (1study), Ethiopia (1study), Zambia (1 study), Congo (1 study), India (1 study), Burkina Faso (1 study), Gambia (1 study), Bangladesh (1 study), Nepal (1study), Brazil (1 study), Australian (1 study), Pacific (1 study), Togo (1 study), USA (1study), and Eswatini (1 study). These studies focused on various aspects of reproductive health care, including maternal health care, family planning, prevention of transition of HIV from mother to child, sexual and reproductive health education, and maternal and infant health (Table 3).

### Synthesis of findings

The synthesis of findings led to the emergence of four themes and 14 subthemes. These Themes included: failure to access all-inclusive and integrated quality services, economic issues, couples' personal preferences, and attitudes, as well as sociocultural considerations to seek reproductive health services (Table 4), which is elaborated in the following part.

**Table 4:** Themes and sub-themes emerged from the analysis

Study references	subtheme	Theme
3,4,6,8,14,15,16,17,18,19,24,26,27,28,29,31	Mismanagement and poor policy making as hindrance	Lack of access to comprehensive and integrated quality services
3,4,18,19,34	Inappropriate access to services	
(3,4,5,6,8.12,14,16,18,19,20,21,25,27.28,29,30,31,32,33)	infrastructure of service delivery system as a barrier(standard logistic equipment)	
(1,3,4,5,6,9,12,14.16.17.18,19,20,27,28,29,32,33)	The framework of human force	
2,3,4,6,9,10,12	financial restrictions	Economic considerations
1,3,4,6,10,12,14,16,18	job commitments as limitation	
1,2,3,5,6,7,8,12,	Men's Knowledge and information	Couples' personal preferences and attitudes
1,3,6,7,8,9,12,14,15,16,18,19	Couples' preferences	
3,6,	Couples' communication	
1,2,3,4,5,6,8,13,14,15,18,19,34	Gender perspectives	Sociocultural considerations to seek reproductive health services
1,3,12,13,16,	dominant culture	
7,10	Religious beliefs	
11,9,2,6,12,16,19	Social norms	
3,6,7,16,18,19	Stigma as a barrier	

#### Failure to access all-inclusive and integrated quality services

Based on the literature review, failure to access all-inclusive and integrated quality services was the main hindrance to male engagement in reproductive health care. The availability of health facilities and service environments, including programs, staff, equipment, and professional behaviors, are effective in the presence of men in reproductive health care. This theme emerged from four subthemes: Mismanagement and poor policy-making as hindrances, Inappropriate access to services, The Infrastructure of the service delivery system as a barrier, and the framework of human force.

##### Mismanagement and poor policy-making as hindrances

Most included studies which explored men's participation in reproductive health have been conducted in developing countries. In developing countries, most policies on various aspects of sexual and reproductive health are focused on women. Multiple studies have reported the neglect of men's participation, contrary to the needs of society, in the planning and macro policy-making in the field of reproductive and sexual health. This sub-theme refers to relevant issues to policy making and planning in reproductive and sexual health programs that prevent men from participating in reproductive health care.

Some articles pointed to the mismanagement during the implementation of reproductive health policies and programs that limit men's participation in reproductive health. Regarding the mismanagement, the following issues were reported in the studies. The men weren't allowed to take part in antenatal care [20, 57]. They were not invited to reproductive health services [20]. Privacy in the design of healthcare facilities was Ignored [17, 31, 41]. Multiple services were offered adjacent to each other [31, 41]. Health system factors banned men's participation in reproductive health [18, 33, 43]. The male reproductive needs didn't address[52]. The presence of a couple together in reproductive health care centers was ignored [17, 33, 38, 39, 43]. Healthcare policymakers didn't receive feedback from health workers [42, 48, 49]. In reproductive health services, the support for men's accommodation was ignored [42]. The men's interaction with the health system was restricted [40]. The participation of men in reproductive health care services wasn't supported [66]. Although men were a decisive agent in reproductive health, they were ignored in reproductive health services [66]. There weren't male-friendly reproductive health services [51]. There wasn't guidance to provide information for men on various aspects of reproductive health [51]. The support for performing instructions accurately was limited [51].

On the other hand, sometimes incomplete and ineffective policymaking has provided the ground for men's non-participation. Ineffective policies on various aspects of reproductive health cause insufficient support for men's participation in reproductive and sexual care. In different studies, this issue has been introduced as one of the obstacles to men's participation in reproductive health care. The Poor policy was described with phrases such as Lack of any target for engaging the men directly [20, 31, 42, 49], lack of emphasis on the presence of men [17, 20], and ignoring of men in the health recommendations [40, 41, 59], ignoring advice and services for men's reproductive health in policy [41], Lack of guidelines and standards for the presence of men in reproductive health [16, 18, 41], Applying personal preferences in management and policy-making [18], Governance of gender roles in reproductive health service [18, 41], lack of guidelines for the mobilization of men [51], Limited interaction of key influential decision-makers in the health sector with the community [51].

We found a gap in translating policies to practice in the literature review, which pointed to poor planning. In the studies, this issue was described with these phrases: lack of education for men about the importance of reproductive health [37, 66], design of maternal and child health programs as a limitation [47], failure to fulfill paternal leave [18], and lack of planning to achieve a comprehensive view of men's participation in health workers [51], lack of training, or no Instructions for male integration into health services [51].

##### Inappropriate access to services

Most of the studies included in this research were conducted in low resources countries. In these countries, transportation infrastructure is often not suitable. The residence of most people is far away from the main road. Sometimes these areas are difficult to pass. People are not able to pay the financial costs related to transportation. Also, most people live in rural areas. They engage in occupations such as agriculture. These people need to travel long distances to access health care. As a result, they may miss an entire day of work. These issues can lead to inappropriate access to reproductive health care. Improper access to services is one of the reasons described in most articles as a constraint on men's participation in reproductive health services. In included studies, Inappropriate access to reproductive health care was described in the following phrases: Reproductive health services take a long time [15, 17, 20, 37, 38, 41-43, 49, 50, 53, 58, 59]. Male clients had fewer opportunities for HIV testing [40, 41]. In health centers, services were provided only in the morning [40, 52]. The distance from home and work to health centers was long [16, 17, 35, 44, 52, 53, 65]. Access to services is not permanent for male participants [31, 40, 41, 53]. Access to the centers is difficult due to poor road infrastructure [16, 30, 34, 42, 53, 58]. Access to some services, such as family planning, was low [44].

##### The infrastructure of the service delivery system as a barrier (standard logistic equipment)

The customer-friendly environment is a crucial point in increasing the client’s participation in reproductive health clinics. Providing a customer-friendly environment, requires attention to the infrastructure. In the review of included studies in the field of infrastructure, issues such as the physical environment, equipment, costs of consumables, and the emotional aspect of the clinic space have been discussed. In all articles, participants described the physical environment of health centers as inappropriate for the presence of men. Included studies reported that counseling rooms in health centers are small. The small space of these rooms limits the privacy of clients [17]. There was no private space for men in the health centers [17, 36, 45, 51]. There wasn't appropriate space for men in the reproductive health clinic [18, 31, 38, 42, 43, 49, 51]. Health centers have few seats. Often, there aren't seats for males to sit on [43].

In addition to the physical infrastructure, sometimes the emotional atmosphere of the clinics and the use of the space to implement care programs are unfriendly to the presence of men. Sometimes they are not welcoming to men in the environment of reproductive health clinics. Most articles have reported that another hindrance for men to participate in reproductive health care was the unfriendly atmosphere of centers, which led to the marginalization of male customers. The unfriendly environment of maternity care centers [30, 36, 45], the unwelcome clinic environment for men [38, 49, 52, 53, 57], the unfavorable feminine structure of healthcare clinics [40, 46, 55, 66], Non-private centers [44], and the unattractive content of services and infrastructure of maternal care centers for men [19] were described by the male participant in the studies.

One of the factors in the availability of accessible healthcare services is accessibility in terms of health supplies and equipment. Lack of equipment hindered men's participation in reproductive health care. In some studies, participants stated that due to the lack of supplements and equipment in the centers [17, 20, 31, 53], the restriction on the choice of available male contraceptive methods [68], the lack of equipment in the health center [31], the deficiency of diagnostic equipment [33], the shortage of medicine or equipment [53], did not participate in reproductive health care.

Plenty of reproductive health services in developing countries are provided free of charge or at a minimal cost. However, several studies have described the costs imposed on participants in reproductive health centers as an obstacle to men's participation in this service. In these studies, participants stated that they rarely referred to reproductive health centers due to fear of soliciting bribes [17], hidden costs for providing equipment [49], paying informal out-of-pocket costs [53], the opportunity costs [50], especially in areas where services were provided free of charge.

##### The framework of human force

Healthcare workers are one of the resources for providing reproductive health care. Healthcare workers provide care, education, and counseling services. However, studies have reported that healthcare workers are one of the barriers to men's participation in reproductive healthcare. This issue can be investigated from different aspects, such as Staff deficiency, negative staff attitude, improper staff behavior, and non-professional providing services.

The sufficient number of healthcare providers in reproductive health clinics is associated with the quality of service delivery. Some studies have reported a shortage of health workers as a hindrance to males participating in reproductive health care. The participants reported limited access to professional staff in remote areas [34]. Shortage of healthcare providers [17, 31, 43, 48], shortage of male staff at reproductive health centers [18, 36], inadequacy of male staff in clinics to train male clients [3, 18], absence of healthcare workers [52, 53], heavy workload of health staff [3] are issues that Challenge male participation in reproductive health services.

In addition to the number of staff, interpersonal communication skills and the attitude of staff towards the presence of men in reproductive health clinics affect the participation of men in reproductive health care. In the reviewed studies, unfriendly attitudes of health care providers were one of the factors preventing men from participating in reproductive health care. The unfriendly attitude of the personnel was described in the studies as negative attitudes towards males participating [3, 18, 30, 36, 42, 43, 49, 53, 60]. Unwelcoming attitude [31], non-acceptance of the presence of men [38], the presence of a male, that embarrasses female staffs [18], indifference to work [53], the potential mistreatment following the health providers negative attitudes [52] were described in studies. Also, participants explained that the mistreatment of healthcare providers towards women and their husbands reduced men's participation in healthcare. Participants described the misbehavior of healthcare workers as the use of heartless language [15, 17], misbehavior [17, 20, 53], unprofessional behavior [31], mistreatment [17, 41], unwelcome behavior [38, 42], rude behavior [17, 40, 44, 53], use of disrespectfullanguage [17, 53, 59], unpleasant behavior [48], use of harsh and sarcastic expressions [49].

In addition to the mistreatment of health care providers, care providance in an unprofessional manner makes men reluctant to participate in reproductive health care. The cases mentioned in the studies that confirm the unprofessional behavior of the health care providers are explained with expressions like a passiverole to provide the correct information [34], failure to state the reason for the importance of men's participation [40], lack of clear justification of advice for men [44], ignoring men’s fear and concern [44, 49].

#### Economic issues

According to the literature review, studies have shown that men's economic status is another barrier to engaging theme in reproductive health care. This theme emerged from two sub-themes: financial restriction and job commitments as a limitation.

##### Financial restriction

The studies reviewed in this study were conducted in developing countries. In these countries, culturally, men are the main factor in decisions related to the health of their family members. Often, decisions about when, where, and how family members access healthcare are made by men. This particular position in decision-making process is related to the role of men as livelihood providers in their families. They are often the main decision-makers regarding the allocation of money for the availability of food, transportation, and costs associated with family members to attend health services. Therefore, as the head of the household, they prefer to being the provider. They prefer to provide necessities for life In these studies, this issue was raised this way. Participants, discussed the challenge of providing necessities for family and participation in reproductive health care [16, 20, 31, 36]. Some participants cited the funding problem as the reason for inactiveparticipation [20].

Participants prefer to focus on economic and income-generating activities [16, 36, 48, 61]. The norms of the community prefer to make money for the family [33]. Traditional health services for mothers were chosen because of financial issues [65]. Financial instabilities inhibited male involvement [42]. Poverty deprived people of the opportunity to choose to participate in PMTCT [35]. The cost of care prevented men from participating in reproductive health care [18, 48]. Concerns about financial support for childcare hinder participation [57]. Also, the lack of insurance coverage for reproductive health services is one of the obstacles to men's participation [40].

##### Job commitments as a limitation

The opportunity cost of men presenting in health service centers instead of attending the workplace plays a significant role in men's decision to participate in reproductive health care. Economic factors affecting men's decision to participate in reproductive health services can be grouped into direct and indirect factors. The direct factors group was explained in the previous sub-theme. The indirect factors have further impact on the decision to attend reproductive health services. In studies, participants discussed the Contrast between job responsibilities with attending reproductive health care for themselves and their spouses. They stated that Job commitments cause a lack of time [17, 31, 36-38, 40, 46, 49, 53, 58, 60, 63, 65]. Leave requests to participate in antenatal care (ANC) from the employer's point is unusual [31, 50, 65]. It is difficult to adjust the time for accompanying the spouses due to work issues [50, 65]. Work takes precedence over accompanying the spouse [36, 46]. Men's working hours interfere with the working hours of care centers [3, 38]. There is a conflict between financing maternal care and leaving work [18, 61, 65]. The male occupation limited spousal support [36, 42].

#### Couples' personal preferences and attitudes

The preferences of couples, especially the male partner, affect men's participation in reproductive health services. According to the results of the present study, beliefs, attitudes, and interactions between spouses and individual factors such as embarrassment, anxiety, distress, and fear caused by inadequate knowledge were the factors that determine men's participation in reproductive health care. This theme explores the preferences of couples and the factors affecting them. This theme emerged from three sub-themes “Men's Knowledge and information”, “Couples’ Preference”, and “Couples' communications”.

##### Men's knowledge and information

Two factors that are important in health behavior are as follows:having health knowledge and access to health information. To increase knowledge, access to information plays an important role. Studies found that inadequate knowledge about reproductive health issues and insufficient information about what is done at the reproductive health centers make men give up accepting their responsibilities to participate in reproductive health care [15-18, 34-36, 44, 48, 52, 54, 71]. Also, inadequate knowledge about the cause of men's participation in maternity services [16, 59] and deficiency of knowledge about the advantages of male participation in health services [31, 55] prevented men from participating in these services. Male participation in reproductive health was influenced by misinformation [32, 44, 55, 64], limited knowledge of the men's role in reproductive services [33, 63], and lack of awareness about the importance of males' engagement in maternity care [18, 34, 36, 58], and misunderstanding of reproductive services [62, 71]. They occur due to men's lack of access to reliable sources of information.

Studies also discussed the cause of the deficiency of knowledge about healthcare. Inadequate family education for men's participation [18, 40], lack of awareness, Poor family performance to encourage men to participate in reproductive health [40], and lack of training for men to participate in reproductive health [18] were the issues raised about lack of awareness. As a result of these issues, it becomes common to follow rumors about reproductive health services. On the other hand, existing knowledge about reproductive health services was not translated into practice [34].

##### Couples preferences

Men's preferences are effective intheir engagement in reproductive health care. Many studies have described male preferences as the reason for inactive participation in reproductive health care. In studies regarding the men's preferences, the expressions of unwillingness to participate in reproductive health care, and education [17, 18, 31, 40, 45], passive attitude to participation in pregnancy care [35, 54], lack of motivation to engage in reproductive health [52], lack of feeling the need to participate in natural childbirth process [16, 36, 39, 58], fear of HIV testing [17, 31, 38, 43, 45, 50, 53], shyness [3, 18, 31, 34, 36, 40, 46, 49, 55, 57, 58, 66], negative attitude towards sexual issues [40], low Perceived risk [40], lack of perceived sensitivity to STDs [40], reluctance to attend a womanly clinic [43], inexperience about participating in maternal care [18], attending reproductive health centers as a time-wasting [49], being concern about privacy [31, 35, 42, 43], being concern about the intentions of health providers [44] were used frequently.

In addition to men's preferences for non-participation in reproductive health care, some studies have reported the reluctance of female partners as one of the reasons for men's non-participation. Some female participants preferred to attend health centers alone. In Studies, the reasons for this issue have been described as feminine embarrassment [30], fear of the husband [30], ignoring the presence of men [45, 63], and trusting their family [18]. Some female participants prevented males from participating in female-related duties [47]. They also did not ask their husbands to participate in reproductive health care [45]. They did not approve of their spouses' decision to have a vasectomy [32].

##### Couples' communications

The joint participation of couples can lead to improve use of reproductive health services. This issue requires communication between couples. The communication between couples helps them to be aware of each other's views on reproductive health services, which leads to mutual understanding. The mutual understanding allows them to agree on shared decision-making about reproductive health services. Inappropriate communication between couples makes men refuse to accompany their wives in reproductive health services. In the studies, expressions such as marital problems between couples [31], predetermined marriage without expressing love [31, 59], lack of interest between spouses [31], lack of consultation between spouses and planning for pregnancy [31], nagging to the spouse while asking him to accompany [31], fear of men's extramarital relationship [41], marital dispute [31, 35], poor relationship between couples [44], mistrust between spouses [18], inappropriate interaction between spouses [18] has been described as factors in preventing male engaging in reproductive health services with their spouses.

#### Sociocultural considerations to seek reproductive health services

The results of the reviewed studies have shown that socio-cultural structures can act as one of the mainbarriers to men's participation in reproductive health services. Also, the results of the studies have shown that people's attitudes toward reproductive health services are influenced by environmental factors such as cultural and social issues. Even more significantly, these attitudes originate from cultural and social issues. As the World Health Organization states, social norms affect families and communities [14]. Therefore, the participation of people, especially men, in reproductive health care is affected by cultural and social factors. This theme discusses sociocultural factors which affect men's participation in reproductive health services. This theme emerged from five sub- themes “Gender perspectives”, “Dominant culture”, “Religious beliefs”, "Social norms", and "Stigma as a barrier".

##### Gender perspectives

Most of the articles reviewed in this study were conducted in African, Asian, and Latin American countries, which are traditional societies. In traditional communities, roles are divided by gender. Also, the dominant culture in these countries is the patriarchal culture. In most studies, gender considerations were described as an effective factor for men's engagement in reproductive health programs. The studies showed that gender duties have traditionally been divided between girls and boys [31, 45, 50, 52, 53]. Male participants described reproductive health as a women's issue [3, 15, 17, 20, 37, 39, 45, 50-52, 60, 64, 66, 71]. Male participants believed that pregnancy, childbirth, and family planning were the responsibility of women [16, 17, 33, 45]. Therefore, women are responsible for the pregnancy and supporting pregnant women [30]. Based on gender role division, men described themselves as producers, provider, administrator [46, 47, 66], and decision-makers [45, 66]. Gender considerations also included the space of reproductive health centers, and maternal and neonatal service centers were described as women's spaces [30, 33]. Studies have shown that gender taboos resulting from gender roles culturally prevent men from participating in reproductive health services [40].

##### Dominant culture

The results of the reviewed studies have shown that the dominant culture of societies was the main factor affecting men's participation in reproductive health services. The culture of the communities where these studies were conducted, does not support male participation in most aspects of reproductive health. Participants stated that their presence in maternity care was contrary to the culture of the community [15]. According to the patriarchal culture in society, men's participation in pregnancy care is unacceptable [18]. Because of Cultural prejudices, men didn't visit medical centers alongwith women [52]. Participants described a negative cultural perception of male participation in reproductive health services [40, 49]. Expressing interest in one's spouse in the community was also considered culturally inappropriate [19]. Culturally, women weren't allowed to ask their husbands to participate in reproductive health services [37]. Men's engagement in maternity care was not culturally accepted [45, 46, 53].

##### Religious beliefs

Religious beliefs are one of the factors that affect men's participation in reproductive health services. Men's participation in some aspects of reproductive health, including family planning and the investigation of sexually transmitted diseases, is most influenced by religious beliefs. In this study, few articles have described religious beliefs in reducing men's presence in the reproductive health program. The use of modern contraceptives, especially vasectomy, was not accepted in some religious sects [32, 44, 55, 64, 65]. Few participants stated that the use of medicine is not according to God's will and contaminates the body of humans which is God's sanctuary. Receiving hospital delivery treatment was described as uncertainty about God's healing power [65].

##### Social norms

Men's participation in reproductive health services is a social and behavioral action. The results of these studies have shown that social norm is one of the barriers to men’s participation in reproductive health services. Attention to social norms in men's decisions to attendreproductive health centers was reported in the studies [16, 33, 36, 46, 50, 51, 54, 66]. Participants said: in their communities, the presence of men in reproductive health centers was unfamiliar [16, 50] and socially unacceptable [66]. Male participants statethat they refused to attend antenatal care due to fear of being seen by community members [36]. Social consensus has limited the presence of men in maternity care and reproductive health care [52]. Some participants described having a crowded family as social credit, so they did not participate in family planning programs [33, 64, 71].Vasectomy was perceived as a factor to therats the role of men as heads of families in society [64].

##### Stigma as a barrier

Social stigma can be scandalous, shameful, and even disgusting and can damage people's social identity.The context of the research investigated in this study is patriarchalIn these societies he presence of men in many aspects of reproductive health is socially and culturally stigmatized, so men did not participate in reproductive health services. The results show that one of the main preventive factors for males' participation in reproductive health was fear of stigma [30-34, 36, 37, 40, 41, 43, 53, 57, 58, 66]. Participants reported that men were ridiculed or humiliated for being involved in maternal care [47]. Fear of other men's reactions, prevented them from participating in various aspects of reproductive health [3, 36, 37]. If men participated in reproductive health programs, they would be described in negative and derogatory terms such as "under the contrl of woman", "waiting like the woman" and "dominated by the woman" [19, 32, 34, 40, 49, 66].

### Line of argument synthesis

Despite emphasizing the importance and benefits of men's active participation in reproductive health programs to the health of men, women, and children, most men are not engaged in reproductive health services. The present study originated from the result’s synthesis of 47 studies conducted on multiple aspects of reproductive health in various contexts. In this study, despite the differences in the context of studies, a significant similarity in the experiences about reasons for not men's involving in reproductive health services was shown. The most important reasons for men's non-participation in reproductive health services, which have been mentioned in different parts of the world, included the failure to access all-inclusive and integrated quality services, economic issues, Couples' personal preferences and attitudes, and sociocultural considerations to seek reproductive health services. These factors are interrelated. Meanwhile, other causes are impressed with cultural and social considerations, such as gender roles and patriarchal culture. Therefore, to achieve the active participation of men in reproductive health services, in addition, to paying attention to these reasons, the relationships between them should be considered. The socio-cultural factors can directly and indirectly affect men's participation in sexual and reproductive health services. So it requires special consideration. It is necessary to try to mobilize agents affecting cultural and social issues, including activists of sociocultural, to provide accessibility of men to comprehensive sexual and reproductive health services.

## Discussion

This meta-synthesis focused on barriers to male participation in reproductive healthcare. It provided a deep insight towards creating a comprehensive synthesis of views of women, men, and healthcare providers concerning barriers to men's involvement in reproductive health. The findings of this synthesis can help policy-making and planning to remove barriers to men's engagement in reproductive health care. Qualitative studies conducted in different countries with different socio-cultural contexts pointed to a group of partially common barriers to male participation in reproductive health services. In this meta-synthesis, four main themes emergedsuch as, failure to access all-inclusive and integrated quality services, economic issues, couples' personal preferences and attitudes, and sociocultural considerations to seek reproductive health services.

### Failure to access all-inclusive and integrated quality services

In the policymaking and managing of reproductive health centers, the concept of male participation in reproductive and sexual health has not been developed yet. In most parts of the world, women are still the primary target of health care services [66]. In line with the results of this study, political and conceptual barriers related to reproductive and sexual health lead to men deprivation of reproductive and sexual health care services [72]. The lack of mutual communication between reproductive health policymakers and service recipients (couples) causes reproductive health policy to be designed and implemented hierarchically from top to down [73]. Mutualinteraction between policymakers, implementers and potential propagandists of reproductive health programs includingreligious leaders and social leaders is the main factor to achieve better results in reproductive and sexual health programs. Lack of clarity of the concept of programs, interaction, and feedback between policymakers, executives, and service recipients leads to disruption of policy implementation [51].

The availability of health facilities and service environments, including programs, staff, equipment, and professional behaviors, are effective in the presence of men in reproductive health care. Although male participation in reproductive health services is encouraged, the status of healthcare facilities for men and couples has not changed [74]. It seems that the existence of obstacles such as unfavorable environments, the unfriendly atmosphere of service centers, and the framework of human force have dissuaded men from actively participating in reproductive health care with their wives [18]. It is recommended that affordable access to reproductive health services should be provided to all, despite gender, race, and socioeconomic status [75]. According to the study findings, there is no specific target for men in reproductive health programs. These studies found that in addressing gender socialization in male adolescents, reproductive health services have ignored the needs of male adolescents and they are unfriendly to men [76].

### Economic issues

Economic status is one of the main structural determinants of perceived equality in the context of reproductive health [76]. Clients' financial limitations were reported as an effective factor in the presence of men in reproductive health centers [77].

Men played the role of the financial supplier of reproductive health care for their families. The high cost of care and the lack of sufficient resources jeopardize the men's role[78]. According to the findings of this study, in addition to financial constraints, job responsibility also is known as an obstacle to the presence of men in reproductive health care. This factor indirectly affects men's economic status. For men with little daily income, leaving the workplace to attend reproductive healthcare can put their economic situation at risk, and it can affect their decision to participate in reproductive health care [79].

### Couples' personal preferences and attitudes

Men's health-seeking behaviors are influenced by some factors, such as embarrassment, anxiety, distress, and fear caused by inadequate awareness about services and medical culture and the prevalence of patriarchal attitudes in men [80]. Men's tendency to participate in reproductive health care is related to their knowledge and attitudes toward reproductive health services [81]. Studies have shown that shamefullness, and reluctance were the main barriers to men's access to reproductive health services such as contraception [82]. Reproductive health is institutionalized as women's health, so providing services in reproductive health centers is not favored by men [72]. Males' unawareness and misperceptions regarding reproductive and sexual health are common barriers for male’s participation in reproductive health [83]. So, promoting the presence of men in reproductive health services requires programs focusing on improving men's knowledge and attitudes using community-based health education programs [84, 85]. Consistent with the results of the current study, beliefs, attitudes, and interactions between spouses are main determinants of male participation in reproductive health care. Poor interaction between the couple is associated with poor men's engagement in reproductive health services [77]. Promoting appropriate couples' interaction about reproductive health services facilitates informed decision-making for spouses [24]. One of the main factors for the presence of men in reproductive and sexual health is realizing the relationship between spouses and identifying the pattern of spouses'relationships regarding reproductive health. Focusing on the marital context of the couples is essential to promote the quality of reproductive health services [86].

### Sociocultural considerations to seek reproductive health services

The findings of a systematic review of the experiences, beliefs, values, and attitudes of adult men about contraception has mentioned that reproductive health-related behaviors affected a person's family, religious, and social contexts which are in confirmity with our findings [24]. In the health promotion programs, socio-cultural subjects that influence health, should be considered. Although personal preferences and attitudes, such as knowledge about reproductive health and communication between spouses, could predict male involvement in reproductive health, cultural subjects, such as the superior power of men in interaction and decision-making, largely determine the presence of men in reproductive health. One of the factors that discouraged men from participating in reproductive health services is the dominant traditional beliefs and gender roles that are culturally determined [87].

Generally, in traditional communities, gender roles have been demarcated to feminine and masculine affairs [18]. There arehuge discrepancies between gender roles. Men's disregard for traditional gender boundaries leads to their ridicule [88]. Regarding health care services, people refuse to seek health servicesdue to fear of stigma. Various aspects of reproductive health care, such as family planning, voluntary sterilization, and physician-assisted reproductive care, can be associated with stigma for users [89]. So, it is necessary to adopt culture-based strategies to improve men’s participation [18]. Therefore, it is necessary to consider the culture of the community in designing reproductive health programs. In designing programs, a couple's attendance should be considered. To educate the community, reproductive health education sessions should be organized in the presence of men and at the community level [52, 53].

There are contradictions in the results of studies on the participation of men in some reproductive health services, such as HIV care. Women believed that their male partner's involvement in HIV care during pregnancy and postpartum, could be beneficial and harmful at the same time.. Men's participation could include a range of support for women and control of their behavior. Gender inequality, along with gender norms in society and HIV-induced stigma, made the situation challenging for women [90]. So, to change the norms of society, action must be taken beyond the health sector to explain policies to protect the rights of men and women on an equal level. Reforms must be aimed at strengthening gender equality so that women can have control over their bodies and lives.Accordingly, every person can decide on his own body without discrimination and compulsion [75]. It is necessary that specialists and their professional associations, which are committed to preserve human dignity, be active to counteract stigmatization among patients and health care providers [89]. To change the norms of patriarchy in society, it is necessary to motivate men to challenge the power and privileges traditionally granted to them. Changing men’s gender attitudes requires short-term interventions, including changing school curricula and forming small groups to create critical thinking about unequal power. It also requires broader social action to change the norm of society, which requires a sociological approach that involves parents and schools in addition to men and consequently community mobilization [91].

Although most of the articles reviewed in the present study were conducted in developing countries, another study also noted the gap and the need for men’s presence while providing health services to women in developed countries. According to the results of this study, Men are almost absent at the time of women's health care and prenatal health education program. Also, programs designed for public health, such as using a social marketing approach, have only targeted women. Men have not been included in the target of "Healthy People 2020 objectives for MCH" [92]. Although interventions to increase men's engagement in mother and infant care have promoted care, there is still a gap in evidence about the efficacy of men's engagement in mother and infant care on morbidity and mortality. Therefore, care should be taken in designing programs to increase men's participation so that their design and implementation can reduce the potentially harmful effects on marital relationships [93].

The current study’s strength was that the investigated articles were qualitative studies that extracted deep information about individuals. The study participants included all individuals involved in reproductive health services, including community members, couples, and health professionals. The study examined reproductive health from various aspects, including maternal health care, family planning, prevention transaction HIV from mother to child, reproductive and sexual health education, and maternal and newborn health. The limitation of the study was that all the articles that were available and reviewed in this study belonged to developing countries.

## Conclusions

The review of studies and their analysis showed that one of the obstacles to men's participation in reproductive health is the lack of access to inclusive and integrated quality services. One of the causes of this lack of access includes mismanagement and weak policies. In the designing and policymaking of reproductive health programs, the position of men as recipients of health services has not yet been determined, which needs to be considered. Men should be included in the goals of policies and plans in reproductive healthprograms. Consequently,the management of reproductive health services, the design of reproductive health environments, and the center's structure of providing reproductive health services became men-friendly. One of the factors that cause men to be left out of reproductive health care and men themselves not want to participate in reproductive health is the dominant culture and social norms, especially gender norms. So gender norms that influence public attitudes toward men’s participation in reproductive health care need to be addressed. To increase men's participation in reproductive health care programs, men’s points of view must be considered. Paying attention to communication skills, especially among reproductive health care professionals, can be an important step in removing barriers to men's participation in these services. The existing gaps in policy making and planning and implementation of programs in men's participation in reproductive health care should be taken into account in conducting future research. Among the factors that are effective in men's participation in reproductive health care are health system programs, policies, economic, and sociocultural attitudes, knowledge, and men's preferences.Therefore, reproductive health initiatives should focus on eliminating challenges to men's supportive activities to incentive men's participation in reproductive health care.

## Data Availability

The datasets analyzed as part of this review are available from the corresponding author on reasonable request.

## Abbreviations

CASP: Critical Appraisal Skills Program
PRISMA: Preferred Reporting Items for Systematic reviews and Meta-Analyses
